# An Authentication and Secure Communication Scheme for In-Vehicle Networks Based on SOME/IP

**DOI:** 10.3390/s22020647

**Published:** 2022-01-14

**Authors:** Bin Ma, Shichun Yang, Zheng Zuo, Bosong Zou, Yaoguang Cao, Xiaoyu Yan, Sida Zhou, Jichong Li

**Affiliations:** 1 School of Transportation Science and Engineering, Beihang University, Beijing 102206, China; mabin_wayne@sina.cn (B.M.); yangshichun@buaa.edu.cn (S.Y.); caoyaoguang@buaa.edu.cn (Y.C.); yanxiaoyu@buaa.edu.cn (X.Y.); zhousida@buaa.edu.cn (S.Z.); ZY1913121@buaa.edu.cn (J.L.); 2 China Software Testing Center, Beijing 100038, China; zoubosong@cstc.org.cn

**Keywords:** in-vehicle network, Ethernet, SOME/IP, security, authentication, key agreement, secret, AEAD

## Abstract

The rapid development of intelligent networked vehicles (ICVs) has brought many positive effects. Unfortunately, connecting to the outside exposes ICVs to security threats. Using secure protocols is an important approach to protect ICVs from hacker attacks and has become a hot research area for vehicle security. However, most of the previous studies were carried out on V2X networks, while those on in-vehicle networks (IVNs) did not involve Ethernet. To this end, oriented to the new IVNs based on Ethernet, we designed an efficient secure scheme, including an authentication scheme using the Scalable Service-Oriented Middleware over IP (SOME/IP) protocol and a secure communication scheme modifying the payload field of the original SOME/IP data frame. The security analysis shows that the designed authentication scheme can provide mutual identity authentication for communicating parties and ensure the confidentiality of the issued temporary session key; the designed authentication and secure communication scheme can resist the common malicious attacks conjointly. The performance experiments based on embedded devices show that the additional overhead introduced by the secure scheme is very limited. The secure scheme proposed in this article can promote the popularization of the SOME/IP protocol in IVNs and contribute to the secure communication of IVNs.

## 1. Introduction

Automotive products, automotive industry, and even the transportation mode of urban areas will significantly engage in technological innovation due to the rapid development of ICVs. Automobiles are converted into complex and intelligent systems since they have integrated increasingly advanced information processing technologies. The quantity of electronic control units (ECUs) in cars has exploded. Some luxury cars are equipped with over 100 ECUs [1]. This has not only led to an increase in the wiring harness and assembly costs, but has also prevented automobiles from completing rapid iterations and thereby keeping pace with the development of information technology. A domain-centralized electrical/electronic architecture (EEA) and a vehicle-centralized EEA will build new in-vehicle communication networks with the in-vehicle Ethernet as the core backbone network. These can provide a higher communication bandwidth for in-vehicle infotainment (IVI), cameras, lidars, and other sensors required for smart driving; decrease the costs of vehicle manufacturing; and accelerate the iteration of automotive software and hardware [2]. The automotive Ethernet includes many protocol clusters, among which the SOME/IP protocol is a very promising application layer protocol and is gradually popularizing in in-vehicle networks [3,4,5].

The rapid development of ICVs has not only created new requirements for innovations in automobile EEA, but also led to serious cybersecurity issues. The number of cyber attacks on automobiles has risen dramatically in this decade. According to the Upstream Security’s report, the record of automotive cyber attacks increased six times from 2016 to 2019, doubling from 2018 to 2019 [6,7]. More than half of these attacks were conducted by hackers rather than security researchers. In total, 57% and 55% of automotive cybersecurity incidents were initiated by cybercriminals in 2019 and 2020, respectively [7,8]. Among them, in 2015, the report of remote hacking into a Jeep Uconnect system resulted in wide concern in the automotive industry. Charlie Miller and Chris Valasek sent illegal commands to control the steering and brake systems by remote attack, which led to the costly recall of 1.4 million vehicles [9,10]. This incident started a new era in automotive cybersecurity. Governments and manufacturers have started to pay increasingly more attention to automotive cybersecurity. The issue has greatly extended from the academic field to the industrial field.

In summary, this article orientates the new in-vehicle network with Ethernet as the core backbone, and studies security protection measures based on applied cryptography technology. The main contributions are as follows.

1. An efficient authentication scheme is designed based on the SOME/IP protocol. The scheme uses a safety and security controller as the key management center (KMC) of the in-vehicle networks and is implemented based on symmetric cryptography. The session keys will be regularly updated and distributed to the domain controllers to resist brute force attacks on the key.
2. A secure communication scheme is designed based on the authentication scheme. The payload field of the SOME/IP data frame is modified to provide integrity and confidentiality protection for the communication process without changing the basic structure of the data frame.
3. Informal and formal security analysis is carried out to evaluate the security of the proposed scheme based on common automotive cyber attacks.
4. An experimental platform including safety and security controller and domain controllers is built to evaluate the performance of the designed secure scheme, including latency, calculation, and system resource overheads.

The rest of the article is organized as follows. Section 2 reviews related work and literature in this field. Section 3 defines the system model, the threat model, and the security goal. Section 4 elaborates the designed secure scheme in detail and the security of the proposed scheme is analyzed in Section 5. Finally, we evaluate the performance of the designed secure scheme quantitatively based on the experiments in Section 6 before concluding the article in Section 7.

## 2. Related Work

In this section, we review the existing studies on secure schemes for in-vehicle networks, secure schemes for V2X networks, and the development of the automotive Ethernet. Automobile cybersecurity protection is a typical interdisciplinary topic. At present, most scholars in the cybersecurity field have backgrounds in computer networks, computer security, mathematics, control algorithms, and cryptography. Research on the cybersecurity of in-vehicle networks involves electronics, embedded systems, and even mechanical fields, and these contents often have a certain degree of heterogeneity in different vehicle models. Therefore, this mismatch of professional backgrounds means that most related studies are conducted on pivotal external interfaces, such as Telematics Box (TBOX), gateways, and IVI. Additionally, current studies on vehicle secure schemes have mostly focused on the field of Vehicle-to-Everything (V2X) networks. However, the studies on secure schemes for in-vehicle networks are becoming more important and more extensive since the development of related technologies ICVs and the training of relevant composite technicians.

The studies in [1,11,12,13,14] focus on authentication schemes for in-vehicle networks. Lu et al. [11] proposed a lightweight encryption and authentication scheme, LEAP, to protect Controller Area Network (CAN) communication. The authentication scheme of LEAP updated the session keys periodically based on the long-term keys and the secure communication scheme used the session keys to avoid the overuse of long-term keys. Mundhenk introduced a lightweight authentication scheme for FlexRay named LASAN in his PhD thesis [1]. The scheme dispersed computing requirements and reduced the latency in real-time scenarios by separating the asymmetric cryptographic component and the symmetric cryptographic component. The simulation results showed that there is one to two orders of magnitude reduction for time delay compared with TLS, TELSA, and Kerberos schemes. In addition, Mundhenk declared a novel risk assessment method based on the Markov model to quantitate the security level of the in-vehicle networks. Agrawal et al. [12] distributed the session key to the ECUs in the CAN with a Flexible Data-Rate (CAN-FD) network based on the symmetric long-term keys. In their work, the gateway was established as the KMC, and the payload of each CAN-FD frame was divided into a 36-bytes message payload and 28-bytes tag. Groza et al. [13] implemented and evaluated four key agreement protocols for CAN-FD bus, including short signatures, identity-based signature, the tripartite Diffie–Hellman key exchange, and identity-based key exchange. Considering that identity-based protocols do not require pre-installed public key certificates during identity authentication and key agreement, the author recommended using identity-based protocols in the in-vehicle network. Jo et al. [14] designed a new authentication protocol for CAN bus, named MAuth-CAN. This protocol can resist the masquerading attack and DoS (Denial of Service) attack of a malicious node without greatly increasing the bus load and modifying the hardware. However, the latency of the protocol needs to be further optimized in the real-time application environment.

The studies of [15,16,17,18] focus on investigating the secure communication scheme for in-vehicle networks. Bella et al. [15] designed a secure communication scheme called LibrA-CAN to build message authentication in CAN communication based on the message-digest algorithm (MD5). This scheme split a CAN message into a normal CAN message with the payload and another additional CAN message with the authentication code (MAC), doubling the overhead of the CAN bus. Carel et al. [16] used the lightweight MAC called Chaskey to provide integrity (without confidentiality) for the CAN-FD communication. This scheme divided the payload of the CAN-FD frame into a 4-bytes counter field, a 16-bytes MAC field, and a 44-bytes payload field. Farag et al. [17] implemented authentication and encryption for the CAN bus in the hardware model. This scheme employed a dedicated controller to manage all ECUs in the CAN bus, and assumed that all ECUs were distributed with the keys during the manufacturing and supported the Physical Unclonable Function (PUF). Kim et al. [18] proposed a secure communication method for CAN bus, which uses a data compression algorithm to reduce the length of the original data filed in the CAN frame. Thus, the data filed would have enough space to accommodate the MAC used to provide message integrity authentication. This method cleverly avoids the modification of the original CAN frame structure but only compresses the data filed to ensure that all CAN frames have MAC authentication with a length of at least four bytes.

Although the studies of [1,11,12,15,16,17,18] have contributed to the in-vehicle networks’ secure schemes, they do not involve the automotive Ethernet, which will be one of the most important communication buses for in-vehicle networks in the future. On the other hand, in the field of protocol conversion between traditional vehicle buses (CAN, CAN-FD, and FlexRay) and automotive Ethernet, researchers have carried out security-related work.

Trong Yen Lee et al. proposed a routing mechanism between Ethernet and FlexRay in [19]. This routing mechanism was integrated into an FPGA gateway system in [20]. The simulation results showed that the gateway system had good latency and power consumption characteristics. Jin Seo Park et al. [21,22] proposed a routing method between Ethernet and CAN/CAN-FD. The routing method was divided into a direct routing mechanism and an indirect routing mechanism according to the integrated message authentication method in the routing process, and the routing performance was measured and evaluated. The results showed that the transmission time of the CAN message from the ECU to the gateway accounts for the largest proportion of time taken in the entire routing process.

Although the studies of [19,20,21,22] involve automotive Ethernet, they focused on protocol conversion with security mechanisms. Moreover, the Ethernet protocol they used does not involve SOME/IP, an application layer protocol that follows Service-Oriented Architecture (SOA). The studies of [19,20] used Transmission Control Protocol (TCP) for Ethernet data forwarding, and the studies of [21,22] used IEEE 1722 for Ethernet data forwarding.

## 3. Preliminary Background and System Models

### 3.1. Preliminary Background

#### 3.1.1. Authenticated Encryption and Associated Data Algorithm

**Figure 1:**
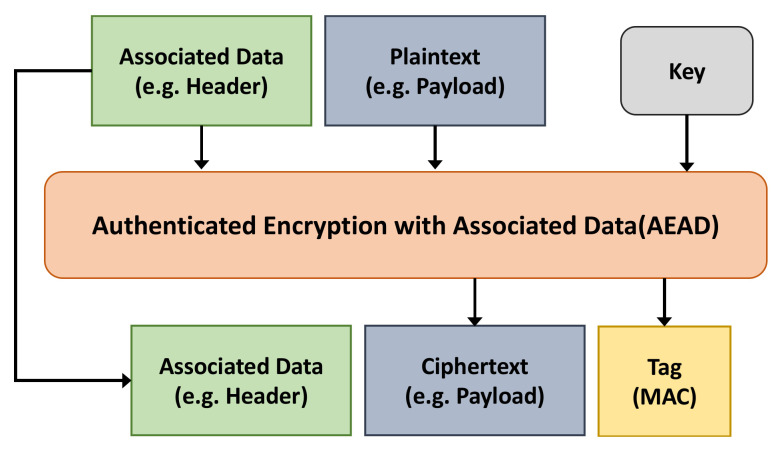
Principle of the AEAD algorithm.

The AEAD algorithm is a cryptographic algorithm that can provide confidentiality and integrity protection at the same time [23]. An example illustrates its working principle, as shown in Figure 1. The message consists of a header field and a payload field. The header contains information, such as the source and destination address and the check digit required for message transmission and paring. The payload is the effective data that needs to be transmitted. AEAD provides confidentiality and integrity protection for the payload to prevent malicious attacks, such as frame sniffing and frame forgery. At the same time, only integrity protection is provided for the header to prevent frame forgery attack while ensuring that the message can be transmitted correctly.

#### 3.1.2. Scalable Service-Oriented Middleware over IP Protocol

The development of automotive Ethernet depends on the standardization work of alliances, such as IEEE, OPEN, AUTOSAR, AVnu, etc. There are three key achievements for the physical layer of automotive Ethernet including BroadR-Reach technology designed by Broadcom, AVB/TSN technology designed by AVnu Alliance, and TTEthernet technology designed by TTTech. Among these, BroadR-Reach has been standardized by the IEEE802.3bw working group and named 100BASE-T1, also called OABR (OPEN Alliance BroadR-Reach) [24]. The automotive Ethernet adopts the IEEE 802.3 interface standard to seamlessly support the widely used TCP/IP protocol cluster without any adaptation. The application layer protocols of automotive Ethernet include SOME/IP [3,4,5], Do/IP [25,26], and Universal Measurement and Calibration Protocol (XCP) [27], etc.

**Figure 2:**
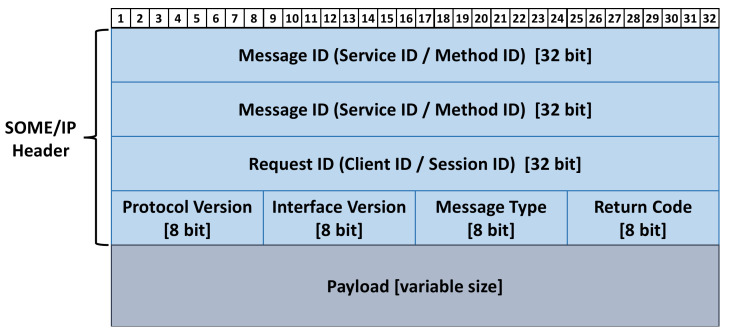
SOME/IP data frame structure.

SOME/IP is a scalable middleware used to transmit service information. It adapts to varied devices with different operating systems, such as cameras, IVI, or autonomous driving modules. SOME/IP is a service-oriented communication technology, which is different from the signal-oriented communication technology as CAN communication. SOME/IP provides three main communication models: Service Discovery (SD), Remote Procedure Calls (RPC), and Publish/Subscribe Mechanism. It does not include any security features that protect applications and transmitted data from malicious attacks, although it is a promising SOA middleware. The data frame structure of SOME/IP is shown in Figure 2.

### 3.2. Network Models

**Figure 3:**
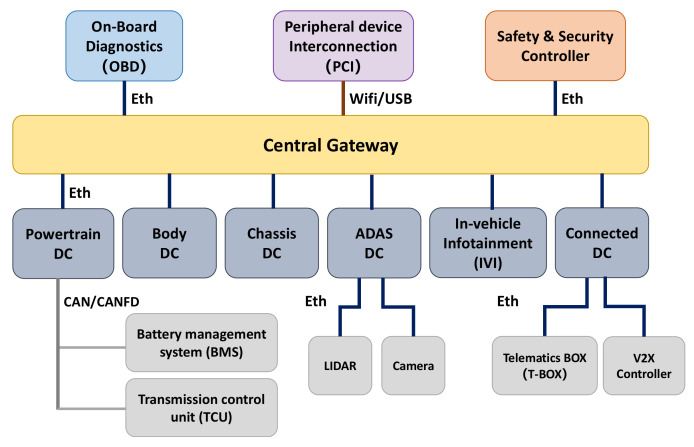
General domain-centralized electrical/electronic architecture.

A general domain-centralized EEA with Ethernet as the backbone bus is shown in Figure 3. The central gateway serves as the in-vehicle information transmission hub and is connected to domain controllers (DCs) through the Ethernet. The relevant ECUs running an Operating System (RTOS) are connected to the powertrain, body, and chassis DCs. The sensors, such as lidar and cameras, are connected to the Advanced Driver Assistance System (ADAS) DC by Ethernet. The T-BOX and V2X controllers are connected to the external networks through wireless communication and connected to the connected DC by Ethernet. The safety and security controller is used to manage functional safety and cybersecurity protection.

The authentication and secure communication schemes are designed for the domain-centralized EEA in this article. The safety and security controller serves as the KMC assist DCs to complete mutual identity authentication and session keys agreement. After that, DCs start secure communication based on the session keys obtained.

The notations used in this article are shown and described in Table 1.

### 3.3. Attack Models

The adversary we consider in this article is attackers that attempt to breach security by monitoring or modifying the messages transmitted in an in-vehicle network. The attack models reported in [9,10,28,29,30,31,32] are considered. These attacks mainly consisted of the following basic attack models:

1. Eavesdropping attack. The attackers intercept the messages transmitted in the communication channel secretly, which make them have the ability to obtain the information of the unencrypted messages or try to decode the encrypted messages.
2. Replay attack. The attacker retransmits the message intercepted from the bus, which will disrupt data flow in the communication channel.
3. Man-in-the-middle attack. The attackers eavesdrop on an existing communication in the middle of two parties and retransmit the messages, which have been tampered.
4. Masquerading attack. The attackers pretend to be legitimate nodes in the communication networks and transmit and receive messages to other legitimate communication parties.

### 3.4. Security Goals and Assumptions

The security goals of the designed authentication and secure communication scheme have the following goals:

1. Before exchanging information, *DC_i_* and *DC_j_* need to complete the mutual identity authentication and the session key agreement with the assistance of *SSC*. The session keys need to be updated periodically to resist the risks of brute force attacks and key leakage.
2. The scheme can provide security protection of integrity and confidentiality for the communication process between *DC_i_* and *DC_j_* based on the session keys.
3. The scheme is capable of resisting the various cyber attacks described in Section 3.2.

This article needs to meet the following assumptions:

1. *SSC* maintains an identity-key pair composed by *DCID_i_* and *K_i,ssc_* for each DC. The attackers could use the *DCID_i_* fraudulently but impossibly obtain the corresponding *K_i,ssc_*, and they are also incapable of inserting a new identity-key pair into *SSC*.
2. The *K_i,ssc_* and *Ks_i,j_* in all DCs are stored properly and obtained impossibility by attackers.
3. The communication channel is insecure. The attackers are able to eavesdrop on all the information transmitted through the channel, and transmit any information through the channel.

## 4. The Proposed Scheme

The proposed secure scheme consists of two parts. One is the authentication scheme and the other is the secure communication scheme.

### 4.1. Authentication Scheme

The proposed authentication scheme is divided into three steps: (A) Initialization, (B) registration, and (C) authentication and key agreement. The step C is implemented based on the SOME/IP protocol.

#### 4.1.1. Initialization Phase

1. Presetting long-term symmetric keys, *LK_i,ssc_* shared between the SSC and the DCs. These long-term symmetric keys will be used in the authentication and session key agreement in subsequent steps. The long-term symmetric keys could be preset when the automobiles or ECUs were produced.
2. Selecting the hash function *H*(). The SSC uses the hash function to generate the session key *SK_i,j_* in subsequent steps. We select the SHA256 algorithm, which has variable length inputs and fixed 32 bytes outputs.
3. Choosing the symmetric encryption algorithm used in the authentication and key agreement phase. We choose the AES256 algorithm, of which the security strength is sufficient to resist the brute-forced attack.

#### 4.1.2. Registration Phase

1. There are five DCs in the general domain-centralized EEA shown in Figure 3. Based on the communication requirements, all these DCs maintain a DCID table including its own DCID and the DCIDs of the ones it needs to communicate with. For example, the DCID table of *DC_1_* contains *DCID_3_* and *DCID_4_* if *DC_1_* just needs to exchange messages with *DC_3_* and *DC_4_*. Additionally, the communication requests from other DCIDs would be regarded as illegal.
2. The SSC maintains an identity-key pair table, which contains the *DCID_i_* of each DC and the long-term symmetric key, *LK_i,ssc_* shared between *SSC* and *DC_i_*. The SSC would be authenticated with the DC in subsequent steps based on the identity-key pairs in this table, and the verification request from an unknown DCID will be regarded as illegal.

**Figure 4:**
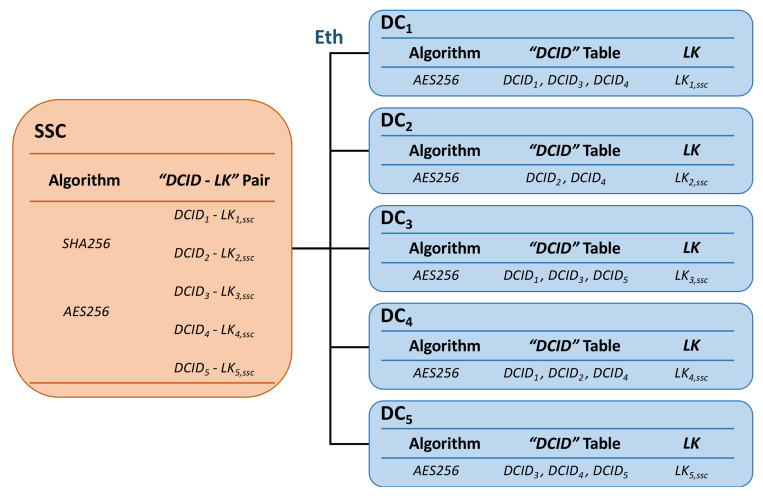
Initialization phase and registration phase.

The description of the initialization phase and the registration phase is shown in Figure 4.

#### 4.1.3. Authentication and Key Agreement Phase

**Figure 5:**
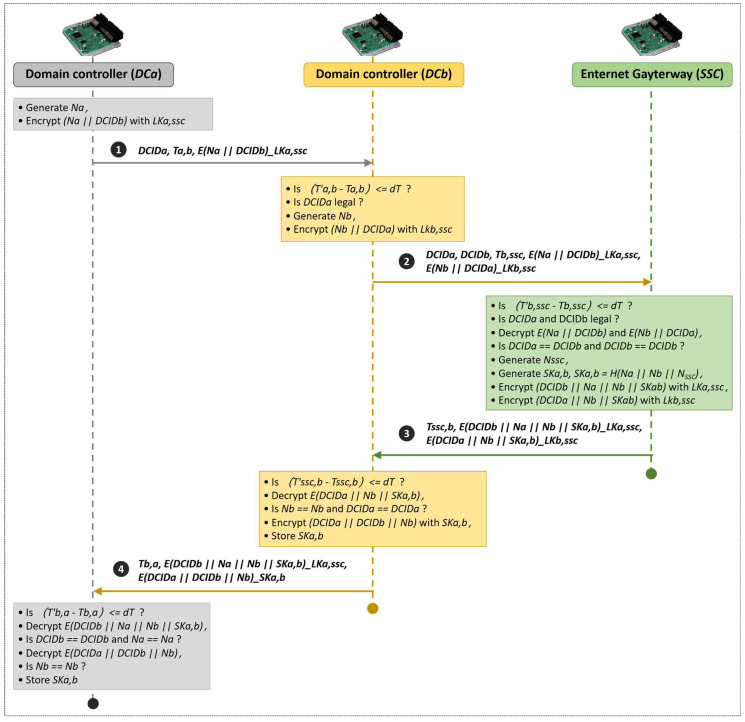
Proposed authentication scheme.

The interaction process of the authentication and key agreement phase is shown in Figure 5. The scheme includes four interactions. The actions that *DC_a_*, *DC_b_*, and *SSC* need to perform during the four interactions are described in detail as follows:

1. Assuming *DC_a_* is the communication initiator that needs to communicate with *DC_b_*. *DC_a_* executes the following steps first:
  - Generating *N_a_* and encrypting the *DCID_b_* of *DC_b_* and *N_a_* with *LK_a,ssc_*. Then, the ciphertext *SENC(N_a_*, *DCID_b_)_LK_a,ssc_* will be obtained.
  - Sending a communication request to *DC_b_*. The request message contains its own ID identification *DCID_a_*, timestamp *T_a,b_*, and ciphertext *SENC(N_a_*, *DCID_b_)_LK_a,ssc_*.
2. *DC_b_* executes the following steps after receiving the communication request from *DC_a_*.
  - Determining whether it is within the allowable range of the time delay between the sending time and the receiving time. *DC_b_* discards the request message if the time delay is unreasonable; otherwise, it continues to the next step.
  - Judging whether *DCID_a_* is legal. *DC_b_* discards the request message if it is illegal. Otherwise, it continues to the next step.
  - Generating *N_b_* and encrypting the *DCID_a_* of *DC_a_* and *N_b_* with *LK_b,ssc_*. Then, the ciphertext *SENC(N_b_*, *DCID_a_)_LK_b,ssc_* will be obtained.
  - Sending a verification request to *SSC*. The request message contains the *DCID_a_* of *DC_a_*, its own *DCID_b_*, timestamp *T_b,ssc_*, ciphertext *SENC(N_a_*, *DC_b_)_LK_a,ssc_*, and ciphertext *SENC(N_b_*, *DCID_a_)_LK_b,ssc_*.
3. *SSC* executes the following steps after receiving the verification request from *DC_b_*:
  - Determining whether it is within the allowable range of the time delay between the sending time and the receiving time. *SSC* discards the request message if the time delay is unreasonable. Otherwise, it continues to the next step.
  - Determining whether the identifiers of *DCID_a_* and *DCID_b_* are legal. *SSC* discards the request message if they are illegal. Otherwise, it continues to execute the next step.
  - Extracting the corresponding long-term symmetric keys *LK_a,ssc_* and *LK_b,ssc_* from the identity-key pair table based on *DCID_a_* and *DCID_b_*. *SSC* uses these two keys to decode the ciphertext *SENC(N_a_, DCID_b_)_LK_a,ssc_* and *SENC(N_b_, DCID_a_)_LK_b,ssc_*, respectively, for obtaining *N_a_*, *DCID_b_*, *N_b_*, and *DCID_a_*.
  - Verifying the consistency of the *DCID_a_* and *DCID_b_* obtained by decryption and the ones obtained by plaintext. The identity verification fails, and the message is discarded if they are inconsistent; otherwise, the process continues to the next step.
  - Generating *N_ssc_*. *SSC* makes SHA256 hash calculation on *N_a_*, *N_b_*, and *N_ssc_* to generate a 32 bits digest *H(N_a_*, *N_b_)*. This digest will be used as the temporary session key *SK_a,b_* between *DCID_a_* and *DCID_b_*.
  - Encrypting *DCID_b_*, *N_a_*, *N_b_*, and *SK_a,b_* with *LK_a,ssc_* for obtaining the ciphertext *SENC(DCID_b_*, *N_a_*, *N_a_*, *SK_a,b_)_LK_a,ssc_*.
  - Encrypting *DCID_a_*, *N_b_*, and *SK_a,b_* with *LK_b,ssc_* for obtaining the ciphertext *SENC(DCID_a_*, *N_b_, SK_a,b_)_LK_b,ssc_*.
  - Returning a response message to *DC_b_*. The message contains the time stamp *T_SSC,b_*, ciphertext *SENC(DCID_b_*, *N_a_*, *N_b_*, *SK_a,b_)_LK_a,ssc_*, and ciphertext *SENC(DCID_a_*, *N_b_*, *SK_a,b_)_LK_b,ssc_*.
4. *DC_b_* executes the following steps after receiving the response message from *SSC*:
  - Determining whether it is within the allowable range of the time delay between the sending time and the receiving time. *DC_b_* discards the response message if the time delay is unreasonable. Otherwise, it continues to the next step.
  - Decrypting the ciphertext *SENC(DCID_a_, N_b_, SK_a,b_)_LK_b,ssc_* with *LK_b,ssc_* to obtain *DCID_a_*, *N_b_*, and *SK_a,b_*.
  - Verifying the consistency of the *DCID_a_* obtained by decryption and the one in the communication request message. The consistency of the *N_b_* obtained by decryption and the one generated by itself is verified. *SK_a,b_* is saved as a temporary session key if the verification is successful.
  - Encrypting *DCID_a_*, *DCID_b_*, and *N_b_* with *SK_a,b_* to obtain the ciphertext *SENC(DCID_a_*, *DCID_b_,N_b_)_SK_a,b_.*
  - Returning a response message to *DC_a_*. The message contains the timestamp *T_b,a_*, ciphertext *SENC(DCID_b_*, *N_a_*, *N_b_*, *SK_a,b_)_LK_a,ssc_* and ciphertext *SENC(DCID_a_*, *DCID_b_*,*N_b_)_SK_a,b_*.
5. *DC_a_* executes the following steps after receiving the response message from *DC_b_*.
  - Determining whether it is within the allowable range of the time delay between the sending time and the receiving time. *DC_a_* discards the response message if the time delay is unreasonable. Otherwise, it continues to the next step.
  - Decrypting the ciphertext *SENC(DCID_b_*, *N_a_*, *N_b_*, *SK_a,b_)_LK_a,ssc_* with *LK_a,ssc_* to obtain *DCID_b_*, *N_a_*, *N_b_*, and *SK_a,b_*.
  - Verifying the consistency of the *DCID_b_* obtained by decryption and the one that is being communicated with. The consistency of the *N_a_* obtained by decryption and the one generated by itself is verified. *DC_a_* continues to the next step if the verification is successful.
  - Decrypting *SENC(DCID_a_, DCID_b_,N_b_)_SK_a,b_* with *SK_a,b_* to obtain *DCID_a_*, *DCID_b_*, and *N_b_*.
  - Verifying the consistency of the two *N_b_* obtained by decryption with *LK_a,ssc_* and *SK_a,b_*, respectively. *SK_a,b_* is saved as a temporary session key if the verification is successful.
6. The temporary session keys *SK_a,b_* between *DC_a_* and *DC_b_* will be updated in the following scenarios by repeating the above five steps:
  - After a preset fixed time period, such as 24 h.
  - After the change of the architecture of the in-vehicle network.
  - When the automobile is starting, idling, or parking.

### 4.2. Secure Communication Scheme

The designed secure communication scheme is implemented based on the SOME/IP protocol. Its core is the modification of the SOME/IP payload field, which can provide security protection without changing the basic structure of the original SOME/IP data frame. This method has also been applied to the design of a CAN/CANFD to SOME/IP gateway by the authors of this article [33]. In this article, the AEAD algorithm is combined with the modification of the SOME/IP payload field to provide integrity and confidentiality protection for the communication process between domain controllers.

**Figure 6:**
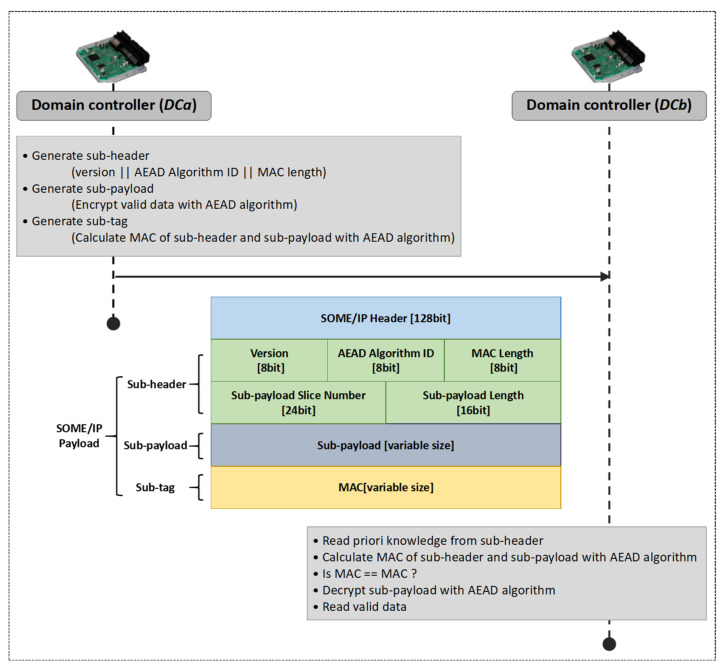
Proposed secure communication scheme.

The communication parties are *DC_a_* and *DC_b_* and the designed secure communication scheme needs to use the temporary session key *SK_a,b_* obtained by the authentication scheme designed in Section 4.1. The description of the designed secure communication scheme is shown in Figure 6.

The sender *DC_a_* modifies the SOME/IP data frame before sending a message. It divides the payload field of the SOME/IP data frame into three portions including a sub-header, sub-message, and sub-tag. Details of the descriptions are as follows:

1. The sub-header contains the information of the protocol version, the type of AEAD algorithm, the length of sub-tag, the number of sub-payload blocks, and the length of the sub-payload block. This information is sent in plaintext to the receiver.
2. *DC_a_* encrypts the effective-load data with the selected AEAD algorithm and the temporary session key *SK_a,b_*. The obtained ciphertext is sent as the sub-payload to provide confidentiality for the communication process.
3. *DC_a_* calculates the MAC of the sub-header and sub-payload with the selected AEAD algorithm and *SK_a,b_*. This MAC is sent as the sub-tag to provide integrity for the communication process.

The receiver *DC_b_* firstly unpacks the received SOME/IP message to obtain the payload. After this, *DC_b_* parses the payload as follows:

1. Reading the protocol version, the type of the AEAD algorithm, the length of sub-tag, the number of sub-payload blocks, and the length of sub-payload block from the sub-header.
2. *DC_b_* calculates the MAC of the sub-header and the sub-payload with the selected AEAD algorithm and *SK_a,b_*. The obtained MAC needs to be compared with the received sub-tag and *DC_b_* will continue to the next step if the two strings are consistent.
3. *DC_b_* decrypts the sub-payload with the selected AEAD algorithm and *SK_a,b_* to obtain the effective-load data in plaintext.

Table 2 shows the AEAD algorithms used in the designed secure communication scheme and the corresponding sub-header field values. AES256-GCM is composed of the AES block encryption algorithm implemented in counter mode and Galois field multiplication [34]. The former provides confidentiality protection, and the latter provides integrity protection. AES56-GCM has 256 bits of security strength and can achieve a higher calculation speed on platforms that support the Advanced Encryption Standard New Instructions (AES-NI). Chacha20-Poly1305 also has 256 bits of security strength and is composed of the Chacha20 stream encryption algorithm and the Poly1305 MAC algorithm [35]. The former provides confidentiality protection, and the latter provides integrity protection. Chacha20-Poly1305 has high performance with software implementation and is suitable for mobile devices without hardware acceleration or AES-NI.

## 5. Security Analysis

The authentication scheme is the premise of the secure communication scheme. In this section, an automatic cryptographic protocol verifier, Proverif, is used to prove the security of the proposed authentication scheme. Then, combined with the threat model proposed in Section 3.2, we conducted an informal security analysis of the overall secure scheme to prove that it can protect the in-vehicle network communication process from various common network attacks.

### 5.1. Security Verification Based on Proverif

**Figure 7:**
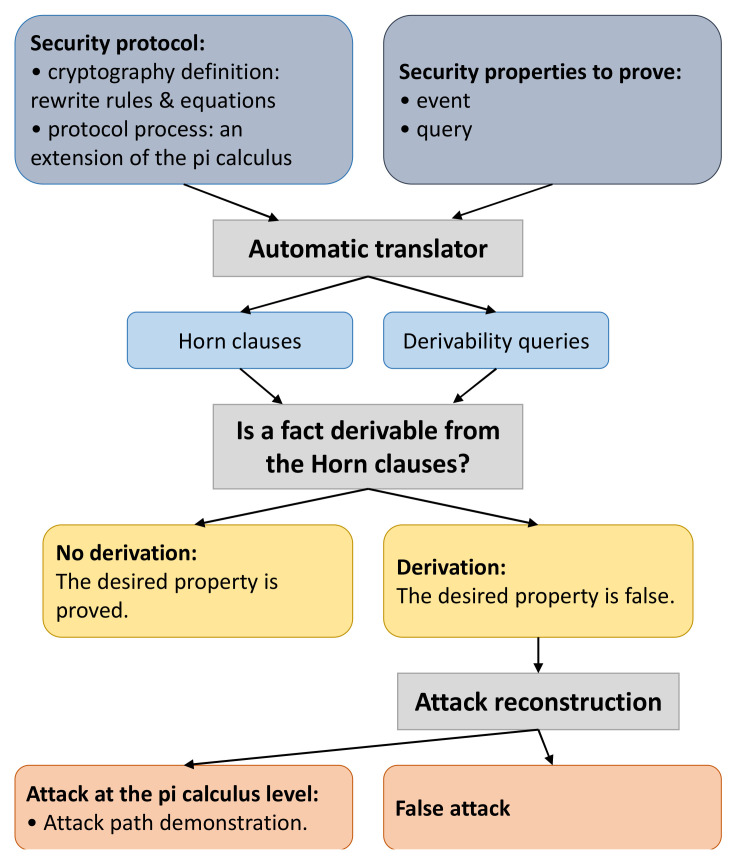
Structure and execution process of Proverif.

Proverif is an automated cryptographic protocol verifier developed and maintained by Blanche et al. The latest version is 2.03. It can prove various security features including secrecy, authenticity, etc. The structure of Proverif is shown in Figure 7, which briefly describes the structure and execution process of Proverif. More details can be found in the literature [36,37].

The input of Proverif includes two aspects, which are the secure scheme and the security features, to prove. The cryptographic algorithms used in the secure scheme are defined by rewrite rules and equations. The execution process of the secure scheme is described in a language similar to the applied pi calculus. The security features to prove are described by a combination of events and queries. After obtaining the required inputs, Proverif converts the secure scheme into a set of Horn clauses and converts the security features to prove derivability queries automatically. Then, Proverif judges whether the security feature represented by a certain query can be derived from the Horn clause based on an internal algorithm. The security feature is proven reasonable if the query cannot be derived. For unsatisfied security features, Proverif attempts to attack the reconstruction. If successful, it will generate feasible attack paths for the secure scheme.

Five processes were built to model the execution process of the designed secure scheme. Process 1, process 2, and process 3 are sub-processes corresponding to the execution processes of *DC_a_*, *DC_b_*, and *SSC*, respectively. Process 4 is used to register the identity-key pair in *SSC* and is a sub-process as well. Process 5 is a main process used to initialize process 1 to process 4. Each execution process is consistent with the description in Figure 5.

Four events were set for characterizing various interactions between the sub-processes.

1. ***event DCA_proved_itself.*** This event is used to record the belief that *DC_a_* completes its identity certification submission. The location of this event is set after the request message has been sent from *DC_a_* to *DC_b_*, that is, after the first message of the authentication scheme.
2. ***event DCB_proved_itself.*** This event is used to record the belief that *DC_b_* completes its identity certification submission. The location of this event is set after the request message has been sent from *DC_b_* to *SSC*, that is, after the second message of the authentication scheme.
3. ***event DCA_accepted_DCB.*** This event is used to record the belief that *DC_a_* believes that it is running a secure scheme with *DC_b_* and has passed the authentication of *DC_b_*. The location of this event is set after the response message has been returned from *DC_b_* to *DC_a_* and has been verified by *DC_a_*, that is, after the fourth message of the authentication scheme.
4. ***event DCB_accepted_DCA.*** This event is used to record the belief that *DC_b_* believes that it is running a secure scheme with *DC_a_* and has passed the authentication of *DC_a_*. The location of this event is set after the response message has been returned from *SSC* to *DC_b_* and has been verified by *DC_b_*, that is, after the third message of the authentication scheme.

Three queries were set for implementing security verification of the designed authentication scheme.

1. ***query attacker(SKAB).*** The actual semantics of this query is *query not attacker (SKAB)*, which is used to query whether the temporary session keys *SK_a,b_* were transmitted by the designed secure scheme without being leaked. It indicates that the designed secure scheme has confidentiality if the query result is true.
2. ***query inj-event(DCA_accepted_DCB) ==> inj-event(DCB_proved_itself).*** The query is used to query that if the *event DCA_accepted_DCB* occurs, then before this event, the *event DCB_proved_itself* must occur. It indicates that *DC_a_* passes the authentication of *DC_b_* in the designed secure scheme if the query result is true.
3. ***query inj-event(DCB_accepted_DCA) ==> inj-event(DCA_proved_itself).*** The query is used to query that if the *event DCB_accepted_DCA* occurs, then before this event, the *event DCA_proved_itself* must occur. It indicates that *DC_b_* passes the authentication of *DC_a_* in the designed secure scheme if the query result is true.

The test results show that all results returned by the above-mentioned queries are *TRUE*. This indicates that the secrecy and authentication of the designed authentication scheme have been verified. *DC_a_* and *DC_b_* have completed mutual authentication and key agreement with the assistance of *SSC*.

### 5.2. Informal Security Analysis

This section conducts an informal security analysis of the proposed security scheme based on the threat models introduced in Section 3.2.

#### 5.2.1. Resist Eavesdropping Attacks

The session key *SK_a,b_* and the nonce *N_a_* and *N_b_* required to generate the session key are encrypted by the AES256 algorithm during the transmission process in the designed secure scheme. It is considered that it is impossible to crack the AES256 encryption algorithm in this article. Hence, attackers are incapable of filching the session key or its components.

The identity *DCID_a_* and *DCID_b_* are transmitted in plaintext in some messages of the designed secure scheme. The attackers are capable of obtaining *DCID_a_* and *DCID_b_* under the premise of knowing the process of the designed secure scheme and the length of each field in the messages. However, it is useless to crack the secure scheme by obtaining the identities only since the authentication and key agreement are completed with the assistance of *SSC*, which works by relying on the identity-key pair maintained internally.

#### 5.2.2. Resist Replay Attacks

Each message transmitted between *SSC*, *DC_a_*, and *DC_b_* contains a timestamp for ensuring the freshness of the message to resist replay attacks.

#### 5.2.3. Resist Man-in-the-Middle and Camouflage Attacks

Man-in-the-middle and camouflage attacks modify the messages transmitted in the channel or generate valid messages directly to deceive nodes. It is assumed that all long-term keys and session keys are stored properly. This means that the attackers are incapable of generating a usable ciphertext contained in a message and incorrect modification of the ciphertexts will cause the execution of the security scheme to fail. Furthermore, although attackers can modify or forge the identities, they cannot cope with the consistency check of the identities in the ciphertext and in the plaintext in the designed security scheme. Specifically, the modification of the identities will also cause the execution of the security scheme to fail.

#### 5.2.4. Provide Mutual Authentication

*SSC* uses the key *LK_a,ssc_* to decrypt the ciphertext *SENC(N_a_, DCID_b_)_LK_a,ssc_* after receiving the verification request message from *DC_b_*—the second message of the designed authentication secure scheme. Then, the consistency of the *DCID_b_* decrypted from the ciphertext and the *DCID_b_* sent in the plaintext will be checked. Since the key *LK_a,ssc_* is only shared between *SSC* and *DC_a_*, passing the consistency verification means that *SSC* has completed the identity authentication of *DC_a_*.

*DC_b_* uses the key *LK_b,ssc_* to decrypt the ciphertext *SENC(DCID_a_, N_b_, SK_a,b_)_LK_b,ssc_* after receiving the response message from *SSC*—the third message of the designed secure scheme. Then, the consistency of the *DCID_a_* decrypted from ciphertext and the *DCID_a_* in the first message of the designed secure scheme, and the consistency of the *N_b_* decrypted from ciphertext and the *N_b_* generated by *DC_b_* itself will be checked. Since the key *LK_b,ssc_* is only shared between *SSC* and *DC_b_*, passing the consistency verification means that *DC_b_* has completed the identity authentication of *SSC* and *DC_a_*.

*DC_a_* uses the key *LK_a,ssc_* to decrypt the ciphertext *SENC(DCID_b_, N_a_, N_b_, SK_a,b_)_LK_a,ssc_* after receiving the response message from *DC_b_*—the fourth message of the designed secure scheme. Then, the consistency of the *DCID_b_* and *N_a_* decrypted from the ciphertext and the *DCID_b_* and *N_a_* in the first message of the designed secure scheme will be checked. Since the key *LK_a,ssc_* is only shared between *SSC* and *DC_a_*, passing the consistency verification means that *DC_a_* has completed the identity authentication of *DC_b_*.

## 6. Performance Evaluation

### 6.1. Experiment Settings

**Figure 8:**
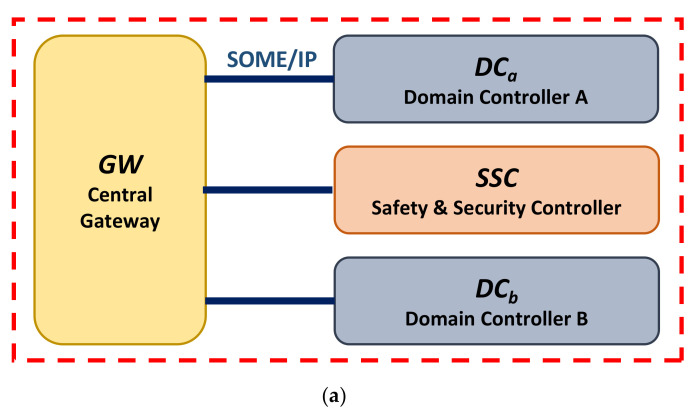

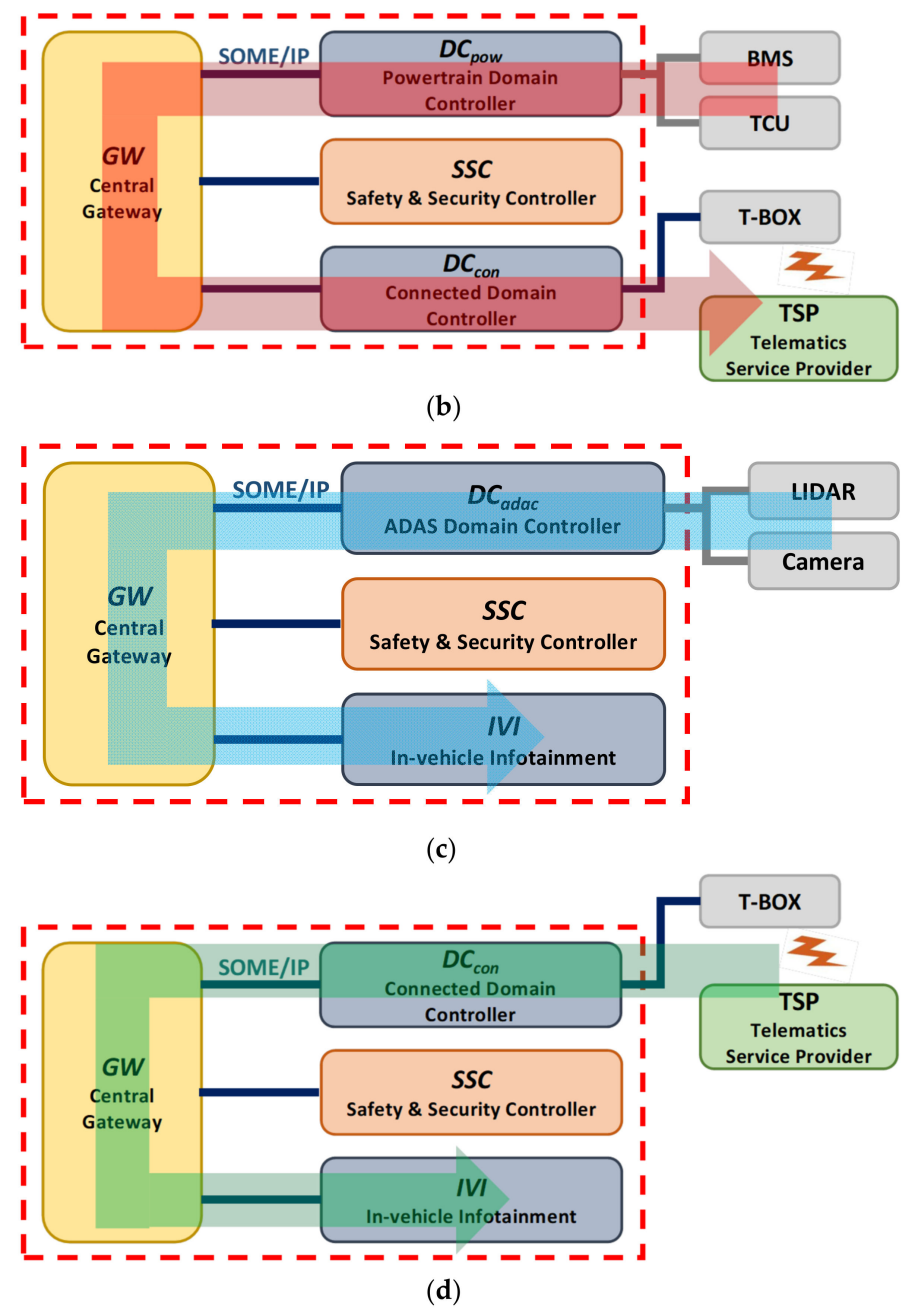
Application scenarios for the experiment evaluation. (**a**) Typical Ethernet-based communication structure. (**b**) Remote Monitoring scenario. (**c**) Parking Assistance scenario. (**d**) OTA upgrade scenario.

Figure 8a depicts a typical Ethernet-based communication structure. *DC_a_* and *DC_b_* complete the mutual identity authentication and session key agreement with the assistance of *SSC*, and then perform secure communication based on the obtained temporary session keys. This communication structure can represent many automotive Ethernet application scenarios, as shown in the red dashed boxes in Figure 8b–d. Figure 8b shows a remote monitoring scenario. *DC_con_* sends a data request to *DC_pdc_* to obtain the powertrain domain data at a certain moment. After *DC_pdc_* returns the data, *DC_con_* sends it to TSP through T-BOX. Usually, the size of the vehicle data collected in this scenario ranges from tens of bytes to several thousand bytes. Figure 8c shows a parking assistance scenario. To provide the driver with a Rear View Camera image or an Aroundview Monitor image, *IVI* sends a data request to *ADAS* to obtain image data from the *Cameras*. This scenario requires continuous transmission of picture files. Usually, the size of each frame of the picture is in the range of dozens of KBytes to several MBytes. Figure 8d shows an OTA upgrade scenario. To complete the system or APP upgrade, *IVI* sends a data request to *DC_con_* to obtain an upgrade package from *TSP*. In this scenario, a software or system upgrade package needs to be transmitted. The package usually ranges from several MBytes to several GBytes depending on the upgrade object.

**Figure 9:**
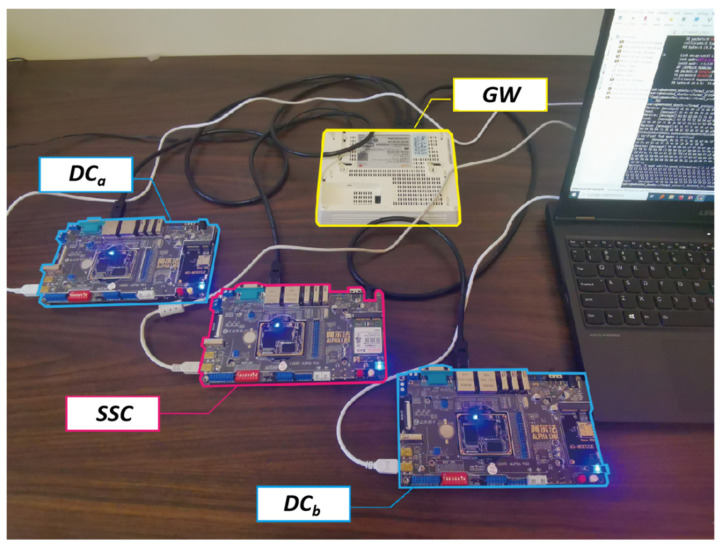
Experimental environment.

The experimental environment is built according to the communication structure shown in Figure 8a. Only the designed secure scheme is implemented without any other functional codes. Thus, the main difference between different application scenarios can be abstracted into the difference of the payload sizes that need to be transmitted. The experimental environment was built based on the above application scenario. *DC_con_*, *DC_pdc_*, and *SSC* were all simulated using the NXP-IMX6ULL boards. Only the designed secure scheme was implemented without any other functional codes in the boards. The IMX6ULL is equipped with Arm Cortex-A7 core clocked at 1000 MHz, and supports 2 CAN channels and a 100 Mbps Ethernet channel. The experimental environment is shown in Figure 9.

The SOME/IP protocol was used in the communication process among *DC_con_*, *DC_pdc_*, and *SSC* and was implemented based on the open-source library of the vsomeip project. Vsomeip is an SOME/IP open-source implementation in the GENIVI project that is based on the Mozilla Public License v2.0 protocol and contributed by BMW. Vsomeip instances communicate with other ECUs through a routing manager that is responsible for the service discovery and the external communication. Multiple vsomeip instances on a same ECU share one routing manager. The first instance started is responsible for starting the routing manager by default. Other instances establish a connection with the routing manager through a routing manage proxy. Furthermore, vsomeip supports local inter-process communication that is implemented through unix socket. A more detailed introduction to vsomeip can be found in the literature [5].

**Figure 10:**
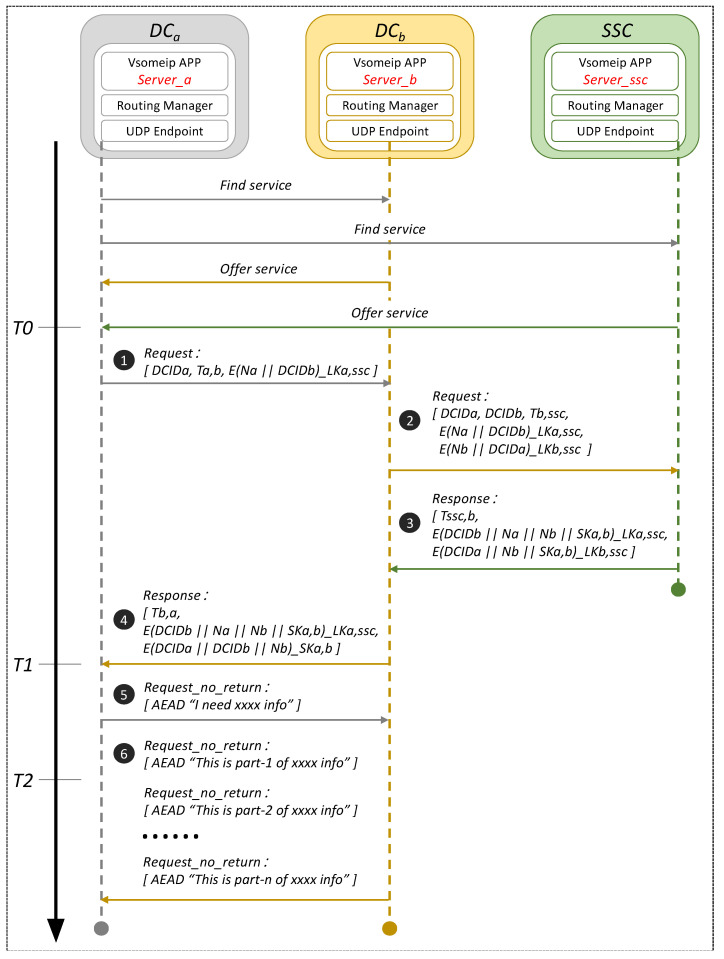
Data transmission process of the experimental program.

The data transmission process of the experimental program is shown in Figure 10. This program includes the authentication scheme and the secure communication scheme designed in this article, and its execution flow completely follows the scheme framework shown in Figure 5 and Figure 6. Some supplementary instructions are as follows.

1. The main bodies of the experiment program are vsomeip APPs in *DC_a_*, *DC_b_*, and *SSC*, named *Server_a*, *Server_b*, and *Server_ssc*, respectively.
2. Before *Server_a* executes the secure scheme, it needs to first determine whether *Server_b* and *Server_ssc* are providing services or not through the service discovery communication model of SOME/IP. Specifically, *server_a* needs to send Find Service messages to *Server_b* and *Server_ssc*, respectively. After receiving the Offer Service messages, *server_a* starts to execute the secure scheme through the remote procedure call communication model of SOME/IP.
3. In order to facilitate performance evaluation, the experimental program includes both the authentication scheme (messages 1–4 in Figure 10) and the secure communication scheme (messages 5–6 in Figure 10). However, in actual application scenarios, the authentication scheme and the secure communication scheme are usually not executed sequentially. They are executed interactively based on the working conditions and may not be executed in the same set of APPs.
4. Messages 1–4 follow the remote procedure calls communication model of SOME/IP, and have a message type of Request/Response. Messages 5–6 also follow the remote procedure called the communication model but have a message type of Request_no_return. When the size of the data to be transmitted is less than a certain value (e.g., 1024 bytes), it is sent as a single message. When the size of the data to be transmitted is larger than a certain value (e.g., 1024 bytes), it is sliced into multiple blocks in units of the certain value for transmission.
5. The three timestamps T0, T1, and T2 in Figure 10 are all collected from *DC_con_*. T0 is the time that *Server_a* finds *Server_b* and *Server_ssc* are providing services and begins to execute the designed authentication scheme. T1 is the time that *Server_a* begins to execute the designed secure communication scheme after completing the mutual authentication and session key agreement with *Server_b*. T2 is the time that *Server_a* and *Server_b* have completed the secure communication.

### 6.2. Performance Evaluation of the Authentication Scheme

#### 6.2.1. Calculation Overhead

The process of authentication and key agreement includes four message interactions as shown in Figure 5. The load overheads of the four messages and the calculation overhead of each controller that participated in the process of message interaction are analyzed gradually.

The load overheads of the designed authentication scheme are shown in Table 3. The lengths of the identities of each DC, the nonce required for generating the session key, the time stamps, and the session key are 1-bit, 8-bits, 4-bits, and 32-bits, respectively. The lengths of the first to fourth messages in the authentication scheme are 14-bits, 24-bits, 94-bits, and 63-bits, respectively, and the sum length of the messages sent during the authentication and session key agreement process is 195-bits.

The calculation overheads of the authentication scheme are shown in Table 4. The low-latency calculations other than encryption, decryption, random number generation, and hashing are ignored. *DC_a_* executes once nonce generation and once symmetric encryption when sending the first message, and twice symmetric decryption when verifying the fourth message. *DC_b_* executes once random number generation and once symmetric encryption when sending the second message, once symmetric decryption when verifying the third message, and once symmetric encryption when sending the fourth message. *SSC* executes twice symmetric decryption when verifying the second message, and once number generation, once hashing, and twice symmetric encryption when sending the third message. The sum of the calculation overhead of the designed authentication scheme is *3T_rand_* + *5T_enc_* + *5T_dec_* + *T_hash_*. Since the lengths of the plaintexts that need to be encrypted are relatively short, the differences of the calculation overheads caused by the length difference and between the encryption and decryption calculation can be ignored. Therefore, the sum calculation overhead is approximately *3T_rand_* + *10T_enc_* + *T_hash_*.

The authentication and key agreement process designed in this article is based on symmetric cryptography and hashing algorithms. The calculation overheads of these two algorithms are much less than that based on the asymmetric cryptographic algorithm. Moreover, the authentication and key agreement are only executed when the automobile is started, idling, or parked. Therefore, it can be considered that the designed authentication scheme only causes low additional load and calculation overheads on the automotive Ethernet communication.

#### 6.2.2. Latency and System Resource Overheads

The proposed secure scheme consists of two parts. One is the authentication scheme, and the other is the secure communication scheme. The goal of the authentication scheme is to complete the mutual identity authentication and session key agreement between the two communicating parties. The lengths of the interaction messages in the authentication scheme are fixed and do not vary with the Ethernet application scenario. 

**Figure 11:**
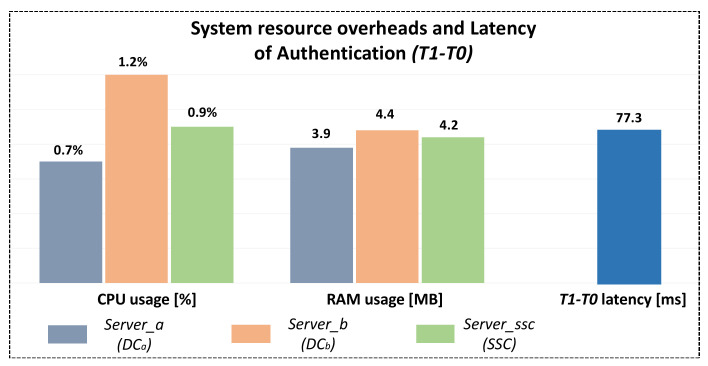
Latency and system resource overheads of the designed authentication scheme.

Three metrics—CPU usage, RAM usage, and latency—were selected to comprehensively evaluate the performance of the designed authentication scheme. During the experiment, the program was modified to make it execute the identity scheme 100 times continuously and abandon the implementation of the secure communication scheme. The CPU and RAM usage of *Server_a*, *Server_b*, and *Server_ssc* were measured, respectively, as well as the overall latency of the authentication scheme (T1-T0 in Figure 10). The average values were taken as the final experimental results, as shown in Figure 11.

It can be seen that *Server_b* consumes the highest system resources. Its CPU and RAM usage are 1.2% and 4.4 MB, respectively. This is mainly because *Server_b* needs to send and receive two SOME/IP messages, respectively, in the designed authentication scheme, while *Server_a* and *Server_ssc* only need to send and receive one message, respectively. The system resource overhead of *Server_ssc* is higher than *Server_a*. This is mainly because *Server_ssc* needs to perform more calculations than *Server_a*. As shown in Table 4, the former is approximately *T_rand_* + *T_hash_* + *4T_enc_*, and the latter is approximately *T_rand_* + *3T_enc_*. *Server_a*, *Server_b*, and *Server_ssc* all consume very low system resources when implementing the authentication scheme. Considering that the IMX6ULL used in our experiment only carries an Arm Cortex-A7 core, it can be believed that the CPU usage will be lower when *Server_a*, *Server_b*, and *Server_ssc* run on some other higher-performance platforms. 

In general, for devices running a Linux operating system, the consumption of system resources by the designed authentication scheme is completely acceptable. Taking into account this low system resource overhead, in actual application scenarios, *Server_ssc* can also be run in a high-performance gateway device or a domain controller instead of separately setting the safety and security controller. In addition, the latency consumed by the designed authentication scheme is 77.3 ms. Considering that the identity authentication and session key agreement are only performed when the vehicle is started, idling, or parking, this latency will not have too much of an impact on the vehicle Ethernet communication.

### 6.3. Performance Evaluation of the Secure Communication Scheme

**Figure 12:**
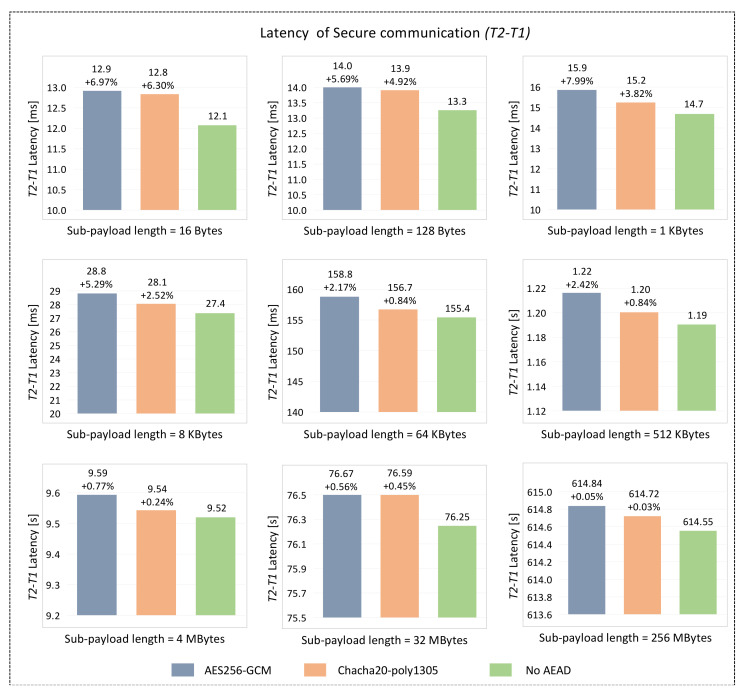
Latency of the designed secure communication scheme.

The goal of the secure communication scheme is to ensure secure transmission of the data. The lengths of the interaction messages in the secure communication scheme vary with the Ethernet application scenario. As mentioned above, different automotive Ethernet application scenarios were simulated by changing the data length returned by *Server_b* to *Server_a*. The effective-load length and the AEAD algorithm selected were used as variables to evaluate the latency characteristics of the designed secure communication scheme. The effective-load length of message 5 in Figure 10 was fixed to 64 Bytes, and no AEAD algorithm was used for encryption and MAC calculation. The effective-load lengths of message 6 in Figure 10 were set to 16 Bytes, 128 Bytes, 1 KBytes, 8 KBytes, 64 KBytes, 512 KBytes, 4 MBytes, 32 Mbytes, and 256 MBytes, respectively. Each message 6 with different lengths was processed using AES-GCM, Chacha20-poly1305, and No AEAD, respectively. Such a data length distribution can cover most automotive Ethernet application scenarios. The experimental results are shown in Figure 12.

It can be seen that compared with the plaintext communication (no AEAD in Figure 12), the additional latency using the AES256-GCM algorithm is between 0.05% and 6.97%, and the additional latency using the Chacha20-poly1305 algorithm is between 0.03% and 6.30%. Ensuring secure communication with Chacha20-poly1305 has a lower additional latency than that with AES256-GCM, while the two algorithms have the same security strength. This is mainly due to the fact that the stream cipher encryption mode of Chacha20-poly1305 is more suitable for embedded and mobile devices. However, the additional latency of AES256-GCM may be lower when it is implemented on some platforms equipped with AES-NI or using hardware acceleration. The latency of the secure communication scheme (T2-T1 in Figure 10) varies with the effective-load length, and its range is 12.1 ms to 614.8 s. It is foreseeable that this value will further increase as the effective-load length increases.

In addition, it is noted that with the increase of the effective-load length, the absolute values of the newly added latency of the AES256-GCM and Chacha20-poly1305 algorithms are increasing, but the percentages are decreasing. The cases with the Chacha20-poly1305 algorithm are taken as an example. When the effective-load length is 16 Bytes, the absolute value of the newly added latency is 0.7 ms, while the percentage is 6.30%. When the effective-load length is 256 MBytes, the absolute value of the newly added latency is 170 ms, while the percentage is only 0.03%. This shows that the latency T2-T1 is mainly determined by the effective-load length and the communication method between *Server_a* and *Server_b*. Although the absolute value of the latency consumed by the AEAD algorithm increases with the increase of the effective-load length, it will be overwhelmed by the latency consumed by the SOME/IP communication model.

In general, the latency difference between the secure communication using the AEAD algorithm and the plaintext communication without the AEAD algorithm is very small. This shows that the encryption and decryption part of the designed secure communication scheme has little effect on the overall latency, and most of the latency comes from the remote procedure calls communication model of SOME/IP.

## 7. Conclusions

An efficient authentication scheme for in-vehicle domain-centralized EEA was designed in this study based on the SOME/IP protocol and symmetric cryptography. A safety and security controller is used as a KMC for in-vehicle communication networks in this scheme. Before communicating with each other, the domain controllers need to complete the mutual identity authentication and the session key agreement with the assistance of the safety and security controller. In order to eliminate the impact on in-vehicle communication as much as possible, the authentication and session key agreement are carried out regularly or when the vehicle is started, idling, or parking.

Based on the authentication scheme, a secure communication scheme was designed to provide integrity and confidentiality protection for the in-vehicle communication process. The payload field of the SOME/IP data frame is divided into three parts—sub-header, sub-payload, and sub-tag. The sub-header contains the information of the protocol version, the type of AEAD algorithm, the sub-tag length, and the sub-payload length. The information provides the message receiver with the prior knowledge required to parse the message. The sub-payload is the ciphertext obtained by using the AEAD algorithm on the effective load, which provides confidentiality protection. The sub-tag is the MAC obtained by using the AEAD algorithm on the sub-header and sub-payload, which provides integrity protection.

The security analysis based on Proverif shows that the designed authentication scheme can provide mutual identity authentication for both communicating parties and can ensure the confidentiality of the temporary session key. An informal security analysis shows that the designed authentication and secure communication scheme can resist common malicious attacks, such as eavesdropping, replay, man-in-the-middle, and camouflage attacks.

The performance evaluation of the designed secure scheme shows that:

1. In the authentication scheme, the total calculation overhead is approximately *3T_rand_* + *10T_enc_* + *T_hash_*, the total latency is 77.3ms, and the consumption of CPU usage and RAM usage by the three communication parties are about 1% and 4MB, respectively. Considering that the authentication and session key agreement are only carried out regularly or when the vehicle is started, idling, or parking, these latency, calculation and system resource overheads will not have too much of an impact on the in-vehicle communication.
2. Most of the latency of the secure communication scheme comes from the remote procedure calls communication model of SOME/IP, and the additional latency caused by the use of AEAD algorithms is very small.

The automotive Ethernet is gradually popularizing in in-vehicle networks, and the SOME/IP protocol is one of the core protocols in automotive Ethernet technology. The secure scheme designed in this article was implemented based on the SOME/IP protocol, which will provide positive support for the promotion of automotive Ethernet and the security protection of in-vehicle networks.

## Figures and Tables

**Table 1:** Notations.

Notation	Description
$DC_{i}$ $,\;DCID_{i}$	$i^{\mathrm{th}}$ DC and its identity
SSC	safety & security controller as the KMC
$N_{i}$	Nonce generated by $i^{\mathrm{th}}$ DC
$N_{ssc}$	Nonce generated by safety & security controller
$T_{A,B}$	The time when device *A* sends message to device *B* (Devices can be $DC_{i}$ or SSC)
${T^{\prime }}_{A,B}$	The time when device *B* gets message from device *B* (Devices can be $DC_{i}$ or SSC)
$LK_{i,ssc}$	Long-term symmetric key shared between $DC_{i}$ and SSC
$SK_{i,j}$	Temporary session key shared between $DC_{i}$ and $DC_{j}$
SENC()_K	Symmetric encryption using K
SDEC()_K	Symmetric decryption using K
H()	Hash calculation
$T_{rand}$	Latency for generating a random number
$T_{enc}$	Latency for performing a symmetric encryption
$T_{dec}$	Latency for performing a symmetric decryption
$T_{hash}$	Latency for performing a hash calculation

**Table 2:** AEAD algorithms and values of the corresponding sub-header.

AEAD Algorithm	AES256-GCM	Chacha20-Poly1305	No AEAD
**Security Strength**	256 bits	256 bits	-
**Version**	0		
**AEAD Algorithm ID**	0	1	2
**MAC Length**	16 Bytes	16 Bytes	0
**Sub-Payload Blocks Number**	variable value		
**Sub-Payload Block Length**	variable value		

**Table 3:** Message length of the authentication scheme.

Item	Length [Byte]
*DCID_a_*, *DCID_b_*	1
*N_a_*, *N_b_*	8
*T_a,b_*, *T_b,ssc_*, *T_ssc,b_*, *T_b,a_*	4
*SK_a,b_*	32
Message 1	14
Message 2	24
Message 3	94
Message 4	63
Sum	195

**Table 4:** Calculation overhead of the authentication scheme.

Electronic Unit	Action	Overhead
*DC_a_*	Send *msg 1*	*T_rand_* + *T_enc_*
	Validate *msg 4*	*2T_dec_*
*DC_b_*	Validate *msg 1*	*-*
	Send *msg 2*	*T_rand_* + *T_enc_*
	Validate *msg 3*	*T_dec_*
	Send *msg 4*	*T_enc_*
*SSC*	Validate *msg 2*	*2T_dec_*
	Send *msg 3*	*T_rand_* + *T_hash_* + *2T_enc_*
Sum	-	*3T_rand_* + *5T_enc_* + *5T_dec_* + *T_hash_* *≈* *3T_rand_* + *10T_enc_* + *T_hash_*

## References

[1] Mundhenk P. (2017). Security for Automotive Electrical/Electronic (E/E) Architectures.

[2] Lock A. Trends of Future E/E-Architectures. https://www.gsaglobal.org/wp-content/uploads/2019/05/Trends-of-Future-EE-Architectures.pdf.

[3] AUTOSAR (2019). Some/Ip Protocol Specification. Autosar Foundation Release R19-11, 696.

[4] AUTOSAR (2019). Some/Ip Service Discovery Protocol Specification. Autosar Foundation Release R19-11, 802.

[5] GENIVI Vsomeip. https://github.com/COVESA/vsomeip.

[6] Upstream Upstream Security Global Automotive Cybersecurity Report 2019. https://upstream.auto/upstream-security-global-automotive-cybersecurity-report-2019/.

[7] Upstream Upstream Security’s 2020 Global Automotive Cybersecurity Report. https://upstream.auto/upstream-security-global-automotive-cybersecurity-report-2020/.

[8] Upstream Upstream Security’s 2021 Global Automotive Cybersecurity Report. https://upstream.auto/2021report/.

[9] Miller C., Valasek C. (2014). A survey of remote automotive attack surfaces. Black Hat USA.

[10] Miller C., Valasek C. (2015). Remote exploitation of an unaltered passenger vehicle. Black Hat USA.

[11] Lu Z., Wang Q., Chen X., Qu G., Lyu Y., Liu Z. LEAP: A lightweight encryption and authentication protocol for in-vehicle communications. Proceedings of the 2019 IEEE Intelligent Transportation Systems Conference (ITSC).

[12] Agrawal M., Huang T., Zhou J., Chang D. (2018). CAN-FD-Sec: Improving Security of CAN-FD Protocol. Proceedings of the Security and Safety Interplay of Intelligent Software Systems, Barcelona, Spain, 6–7 September 2018.

[13] Groza B., Murvay P.-S. (2019). Identity-Based Key Exchange on In-Vehicle Networks: CAN-FD & FlexRay. Sensors.

[14] Jo H.J., Kim J.H., Choi H.-Y., Choi W., Lee D.H., Lee I. (2019). MAuth-CAN: Masquerade-Attack-Proof authentication for in-vehicle networks. IEEE Trans. Veh. Technol..

[15] Bella G., Biondi P., Costantino G., Matteucci I. TOUCAN: A protocol to secure Controller Area Network. Proceedings of the ACM Workshop on Automotive Cybersecurity.

[16] Carel G., Isshiki R., Kusaka T., Nogami Y., Araki S. Design of a Message Authentication Protocol for CAN FD Based on Chaskey Lightweight MAC. Proceedings of the 2018 Sixth International Symposium on Computing and Networking Workshops (CANDARW).

[17] Siddiqui A.S., Gui Y., Plusquellic J., Saqib F. Secure communication over CANBus. Proceedings of the 2017 IEEE 60th International Midwest Symposium on Circuits and Systems (MWSCAS).

[18] Kim Y.-J., Woo S., Chung J.-G. (2021). Triple ID Flexible MAC for Can Security Improvement. IEEE Access.

[19] Lee T., Liao R., Lin I., Tsai J. A Novel FlexRay/Ethernet Gateway for In-Vehicle Networks. Proceedings of the 2019 8th International Conference on Innovation, Communication and Engineering (ICICE).

[20] Lee T.-Y., Lin I.-A., Liao R.-H. (2020). Design of a FlexRay/Ethernet Gateway and Security Mechanism for In-Vehicle Networks. Sensors.

[21] Park J.S., Kim D.H., Suh I.H. (2021). Design and Implementation of Security Function According to Routing Method in Automotive Gateway. Int. J. Automot. Technol..

[22] Park J.S., Heurtefeux K., Eom S., Kim D. Routing Methods Considering Security and Real-Time of Vehicle Gateway System. Proceedings of the WCX SAE World Congress Experience.

[23] Forouzan B.A., Mukhopadhyay D. (2015). Cryptography and Network Security.

[24] (2018). ISO/EC/IEEE International Standard—Part 3: Standard for Ethernet—Amendment 1: Physical Layer Specifications and Management Parameters for 100 Mb/S Operation over a Single Balanced Twisted Pair Cable (100base-T1).

[25] (2011). Road Vehicles—Diagnostic Communication over Internet Protocol (Doip)—Part 1: General Information and Use Case Definition.

[26] (2012). Road Vehicles—Diagnostic Communication over Internet Protocol (Doip)—Part 2: Transport Protocol and Network Layer Services.

[27] Patzer A., Zaiser R. (2016). Xcp–the Standard Protocol for Ecu Development.

[28] Woo S., Jo H.J., Kim I.S., Lee D.H. (2016). A practical security architecture for in-vehicle CAN-FD. IEEE Trans. Intell. Transp. Syst..

[29] Cho K.-T., Shin K.G. Error handling of in-vehicle networks makes them vulnerable. Proceedings of the 2016 ACM SIGSAC Conference on Computer and Communications Security.

[30] Khan J. Vehicle network security testing. Proceedings of the 2017 Third International Conference on Sensing, Signal Processing and Security (ICSSS).

[31] Marchetti M., Stabili D. Anomaly detection of CAN bus messages through analysis of ID sequences. Proceedings of the 2017 IEEE Intelligent Vehicles Symposium (IV).

[32] Iehira K., Inoue H., Ishida K. Spoofing attack using bus-off attacks against a specific ECU of the CAN bus. Proceedings of the 2018 15th IEEE Annual Consumer Communications & Networking Conference (CCNC).

[33] Zuo Z., Yang S., Ma B., Zou B., Cao Y., Li Q., Zhou S., Li J. (2021). Design of a CANFD to SOME/IP Gateway Considering Security for In-Vehicle Networks. Sensors.

[34] Dworkin M. Recommendation for Block Cipher Modes of Operation: Galois/Counter Mode (GCM) for Confidentiality and Authentication.

[35] Langley A., Chang W., Mavrogiannopoulos N., Strombergson J., Josefsson S. ChaCha20-Poly1305 Cipher Suites for Transport Layer Security (TLS). https://www.hjp.at/doc/rfc/rfc7905.html.

[36] Blanchet B. ProVerif: Cryptographic Protocol Verifier in the Formal Model. https://bblanche.gitlabpages.inria.fr/proverif/.

[37] Blanchet B. (2016). Modeling and verifying security protocols with the applied pi calculus and ProVerif. Found. Trends® Priv. Secur..

